# Biological Potential and Medical Use of Secondary Metabolites

**DOI:** 10.3390/medicines6020066

**Published:** 2019-06-12

**Authors:** Ana M. L. Seca, Diana C. G. A. Pinto

**Affiliations:** 1 cE3c-Centre for Ecology, Evolution and Environmental Changes/Azorean Biodiversity Group, University of Azores, Rua Mãe de Deus, 9501-801 Ponta Delgada, Portugal; 2 QOPNA & LAQV-REQUIMTE, University of Aveiro, 3810-193 Aveiro, Portugal

**Keywords:** secondary metabolites, biological activities, medicinal applications, plants, seaweeds

## Abstract

This *Medicines* special issue focuses on the great potential of secondary metabolites for therapeutic applications. The special issue contains 16 articles reporting relevant experimental results and overviews of bioactive secondary metabolites. Their biological effects and new methodologies that improve the lead compounds’ synthesis were also discussed. We would like to thank all 83 authors, from all over the world, for their valuable contributions to this special issue.

This editorial is an introduction to the special issue “Biological Potential and Medical Use of Secondary Metabolites” and contains an overview on the role of secondary metabolites as medicines. In fact, secondary metabolites, used as a single compound or as a mixture, are medicines that can be effective and safe even when synthetic drugs fail. They may even potentiate or synergize the effects of other compounds in the medicine. The research and review articles published in this special issue highlight the secondary metabolites with greater potential for therapeutic application as well as new sources of secondary metabolites well known for their therapeutic properties. The manuscripts published in this special issue are also a showcase of the different methodologies and approaches that researchers use to evaluate, demonstrate, and enhance the properties of secondary metabolites extracted from natural sources including terrestrial plants, marine species, and fungi species such as mushrooms. 

*Ocimum sanctum* L. (according to the “The Plant List” database, this name is a synonym of *Ocimum tenuiflorum* L.), is an Ayurvedic herb of Southeast Asia with a long history of traditional use to treat cough, respiratory disorders, poisoning, impotence, and arthritis [1] and with great chemopreventive and therapeutic potential. Flegkas et al. [2] isolate several secondary metabolites from different classes (four terpenoids, four phenolic derivatives, three flavonoids, two lignans, and one sterol) using chromatographic techniques and elucidate their structures using spectroscopic methods. They also report the interesting proapoptotic and selective activity displayed using (-)-rabdosiin, a tetramer composed of a lignan skeleton connected to two caffeic acids, against MCF-7, SKBR3, and HCT-116 cancer cell lines [2], suggesting this secondary metabolite to be a leading central structure in the development of anticancer drugs.

Malaria continues to be a disease without much effective treatment because of the appearance of mechanisms of resistance to current drugs, so the development of new antimalarial drugs is an important area of research. Based on previous knowledge about antiplasmodial activity against a chloroquinone-sensitive strain of *Plasmodium falciparum* of sargahydroquinoic acid, the main metabolite of brown alga *Sargassum incisifolium* (Turner) C. Aggard, Munedzimwe et al. [3] converted this meroditerpene into several derivatives using semi-synthesis to look for more active derivatives. Ten sargahydroquinoic acid derivatives were assessed regarding their antiplasmodial activity and to explore some structure–activity relationships. The results show that sarganaphthoquinoic acid and sargaquinoic acid are the most promising selective antiplasmodial derivatives. Additionally, the presence of a quinone and carboxylic acid were important for selective activity against the chloroquine-resistant Gambian FCR-3 strain of *P. falciparum* [3].

Several secondary metabolites isolated from the same seaweed, *Sargassum incisifolium*, and some semisynthetic derivatives were tested to evaluate their potential as modulators of inflammatory bowel diseases, such as Crohn’s disease and ulcerate colitis, using various in vitro assays [4]. In fact, inflammatory bowel diseases have become a global health challenge since conventional treatments exhibit moderate efficacy and have significant side effects. The natural compound sargahydroquinoic acid was identified as a promising lead compound due to its effects on various therapeutic targets relevant to inflammatory bowel diseases treatment. Conversion of sargahydroquinoic acid to sarganaphthoquinoic acid greatly improved the peroxisome proliferator activated receptor gamma (PPAR-γ) activity, but this structural modification significantly decreased its antioxidant activity and had a minimal effect on cytotoxicity against a HeLa cancer cell line [4].

Artemisinin is a sesquiterpene lactone compound with a unique chemical structure derived from the sweet wormwood plant, *Artemisia annua* L. It is a very successful clinical drug used in the treatment of malaria [5], and now has a second life as an antitumor agent [6]. Therefore, there is a great demand for new sources of artemisinin, in particular among another *Artemisia* species. Furthermore, since the biotransformation and accumulation of artemisinin depends on the natural conditions, such as light intensity, Numonov et al. [7] evaluated the content of the artemisinin in eight *Artemisia* species collected in Tajikistan, a country with a relatively large number of sunny days per year. The artemisinin content on *Artemisia* hexane extracts, prepared using ultrasound-assisted extraction, was determinate using HPLC. The highest content found, in this study, was in *Artemisia vachanica* Krasch. ex Poljakov (0.34% of dried plant), a new source of artemisinin, and the species with the second-highest content after *Artemisia annua* (0.45 %), while *Artemisia leucotricha* Krasch. ex Ladygina (according to the “The Plant List” database, this name is a synonym of *Seriphidium leucotrichum* (Krasch.) Y.R.Ling.) was the only one in which no artemisinin was detected. The same work shows that the treatment of *Artemisia annua* hexane extract with silica gel as an adsorbent resulted in the enrichment of artemisinin [7].

Pristimerin and tingenone belong to the class of quinonemethide triterpenoids, known as celastroloids, a relatively small class of compounds that exhibit interesting biological activities, such as cytotoxicity and anti-inflammatory, antimicrobial, and antioxidant properties, and accumulate mainly in the root of *Celastraceae* species. Taking into account the chemotaxonomic and therapeutic relevance of quinonemethide triterpenoids like pristimerin and tingenone, Taddeo et al. [8] developed an analytical method for its identification and quantification in the root of species of *Maytenus chiapensis* Lundell. These authors suggest the use of RP HPLC-PDA for the analysis of n-hexane-Et_2_O extract (1:1), the ideal solvent for extraction of these two bioactive secondary metabolites. The proposed method is useful in the analysis of other species of *Celastraceae* and in the analysis of commercial samples [8].

The *Boswellia* sp. are resiniferous trees and shrubs that produce oleo-gum resin, well known as frankincense [9], a natural product of high commercial value used in traditional medicine, religious ceremonies, and cosmetic and perfumery products [10]. Byler and Setzer [11] identified the biomolecular targets docked by some frankincense secondary metabolites using reverse docking analysis, showing that some diterpenes exhibited selective docking to bacterial protein targets and to acetylcholinesterase, while some triterpenoids targeted specific antineoplastic molecular targets, diabetes-relevant targets, and protein targets involved in inflammatory processes. Several medicinal properties of frankincense were corroborated by the molecular docking properties of their di- and triterpenoids. This study opens the way for further investigations of the biomolecular targets identified in this work regarding the improvement of new inhibitors to be used in the treatment of bacterial infections, and inflammatory, diabetes, and Alzheimer’s diseases.

Quy and Xuan [12] used a more traditional approach to suggest cordycepin identified in the mushroom *Cordyceps militaris* (L.) Link ethyl acetate extract as the responsible agent for the extract´s xanthine oxidase inhibitory activity. Using the bio-guided assays approach, they identified the constituents of the most active fractions using GC-MS. They revealed that the fungus *Cordyceps militaris*, used in traditional medicine, is a potential source of cordycepin, the largest constituent of the fraction exhibiting the highest anti-xanthine oxidase effect. Thus, the *Cordyceps militaris* fractions and/or its constituent cordycepin could be beneficial for hyperuricemia treatment. However, more in depth studies and in vivo trials on compounds purified from this medicinal fungus are needed.

Polyphenols are a vast and heterogeneous set of secondary metabolites that include flavonoids, stilbenes, lignans, benzoic acid derivatives, and cinnamic acids, among others, which have in common at least one hydroxylated aromatic ring. They are the subject of vast research as they possess biological properties relevant to well-being and improved health [13,14,15]. In fact, it is known that the consumption of specific types of food (e.g., fruits) rich in polyphenols exerts a positive effect on health, improving, for example, the antioxidant and anti-inflammatory responses of the organism and helps fight cardiovascular and cancer diseases [13,16]. The antioxidant potential and total polyphenols content in most of the 17 ancient regional varieties of apples from the province of Siena in Tuscany are remarkably higher when compared with two commercial varieties, being in some cases about 8 times higher. In addition, older varieties showed lower glucose contents and higher contents of xylitol and pectins, which are also relevant factors for considering older varieties with the highest potential as nutraceuticals [17].

The polar extracts of *Glycyrrhiza glabra* L., *Paeonia lactiflora* Pall., and *Eriobotrya japonica* (Thunb.) Lindl., three known species frequently used in traditional Chinese medicine, were analysed using LC-MS and their total phenolic contents, and antioxidant, antimicrobial, and cytotoxic activities, were evaluated [18]. The terpenoid glycosides was the most abundant class in all three species. Glycyrrhizic acid and (iso)liquiritin apioside isomers were the most abundant secondary metabolites in the *Glycyrrhiza glabra*, while in the *Paeonia lactiflora*, the most abundant were paeoniflorin derivatives, and in *Eriobotrya japonica*, the most abundant were the nerolidol derivatives. The *Paeonia lactiflora* extract was the most antioxidant one, which was more active than the (-)-epigallocatechin gallate positive control [18].

The defensins are a family of cysteine-rich peptides with ≈29–42 amino acids, that play a very important role in the defense system of plants, insects, animals, and humans against invasion by microorganisms. Many of these peptides have been proposed as novel natural antibiotics with great potential for application toward human health and agriculture [19,20]. In fact, due to the increase in the phenomena of resistance to conventional antibiotics, the development of new classes of drugs to combat infections by microorganisms has intensified, with defensins being one of those classes that has gained prominence. Ishaq et al. [21] present the most current overview of the plant defensins applications in the treatment of human infections by viruses, bacteria, and fungi; treatment of hemorrhoids, liver disorders, and cancer; and its use in agriculture as a way to increase agricultural production using natural compounds as phytosanitary agents.

*Cannabis* species contain more than 545 secondary metabolites of different classes but they are chiefly known to possess a great structural diversity of non-nitrogen compounds capable of interfering with the central nervous system, known as cannabinoids, which also exhibit very interesting pharmaceutical properties [22]. The increasing interest of patients regarding the medicinal use of *Cannabis* has been accompanied by a renewed interest of scientists in the potential medical use of various constituents of this plant [22,23]. The review of the literature on cannabinoids identified in *Cannabis* and their application for therapeutic purposes, on the evaluation of its toxicological effects, and the development and improvement of new methodologies for its detection and quantification presented by Gonçalves et al. [24] is of great interest. It opens new lines of research in order to increasingly distinguish the recreational use of the medicinal use of both herbal products derived from *Cannabis* and its secondary metabolites.

Like *Cannabis*, kratom (*Mitragyna speciosa* (Korth.) Havil.) is a species that is also used for medical purposes as an analgesic, and for social and recreational use, being a source of psychoactive agents, mainly alkaloids, and a cheap alternative to opiate-rich substances [25]. The most recent review of the literature on *Mitragyna speciosa* [26] presents the state of the art for its major secondary metabolites, the potential beneficial and toxicological effects derived from its use, and the methodologies for its detection in plant and biological samples. It is concluded that the use of kratom or its metabolites may cause dependence; increase blood pressure; cause liver, renal, and neuronal toxicity; emphysema; excess alveoli inflammation; and even death. On the other hand, kratom has interesting effects, namely antinociceptive, anti-inflammatory, gastrointestinal, antidepressant, antioxidant, and antibacterial properties [26]. However, further studies are required to support the use of the species or its secondary metabolites for clinical purposes.

Tavares and Seca [27] demonstrate how *Juniperus* species are a good bet as a source of secondary metabolites by presenting a review about diterpenes, flavonoids, and one lignan identified in *Juniperus* as having a high potential for the development of new antitumor, antibacterial, and antiviral drugs. Deoxypodophyllotoxin appears to be the most promising lead compound since it has reported antitumor effects against breast cancer acquired resistant cells (MCF-7/A), with a very interesting IC_50_ value in the nanomolar level. The dehydroabietic acid methyl ester derivative, with the substituent (2-(4-(3-(*tert*-butoxycarbonylamino)phenyl)-1*H*-1,2,3-triazol-1-yl)acetamido) at C-14, also seems to be an excellent leader compound since it has shown IC_50_ values between 0.7–1.2 µM against PC-3, SK-OV-3, MCF-7, and MDA-MB-231 tumour cell lines, which is an activity higher than the one exhibited by the anticancer agent 5-FU used clinically.

The *Scabiosa* genus, despite the great controversy regarding the taxonomic classification of its species, is widely considered to be valuable in traditional medicine and the biological potential of its secondary metabolites as effective agents in the treatment of various diseases is well known [28]. Pinto et al. [28] present an update on the information about flavonoids, iridoids, and saponins from *Scabiosa* species that can be highlighted both from the point of view of their biological properties and from the in vivo assays already performed. In fact, these secondary metabolites exhibit interesting effects, such as anti-inflammatory and antitumoral activities, effects that validate and extend some traditional uses of *Scabiosa* species, as well as inspire the development of new drugs based on extracts or pure secondary metabolites. On the other hand, this review also demonstrates that the phytochemistry of several *Scabiosa* species has been neglected. These findings should encourage further studies that can reveal the medicinal potential of this species.

An essential oil is a complex mixture of volatile compounds that exhibit the ability to control the infectious/parasitic diseases, which is a great continuing challenge for global health. In fact, essential oils could exhibit a dual role, being able to control vectors, important in the cycle of disease transmission, and they exhibit relevant activity against the pathogens [29]. However, the solubility and stability of essential oils poses significant problems in the formulation of new products for both vector and parasite control. Echeverría and Albuquerque [30] review several studies related to the development of nanoemulsions containing essential oils as effective formulations to control diseases in humans and animals, since they have lower cost and ecological toxicity. The authors emphasize these formulations as water-soluble and stable alternatives, able to act as larvicides, insecticides, repellents, and acaricides, as well as having antiparasitic properties, such that they have proved to be very efficient in the treatment and prevention of infectious and parasitic diseases. In addition, the nanoemulsion formulation of essential oils makes this pesticide more environmentally friendly [30].

The use of bioinformatics and omic workflow is a very recent approach in the effort to discover natural products in various environments, such as soils, aquatic environments, and microbial communities. Chen et al. [31] present a literature review highlighting several methods, mainly bioinformatics, used to identify biosynthetic gene clusters that encode the biosynthesis of secondary metabolites in the environment, especially in environments where microorganisms are rarely cultivated. There are also several examples of how recent studies have explored the genetic basis for the synthesis of new natural products that have broad medical and industrial applications [31].

By considering all the information given in this special issue, one can confirm the importance of plants in the development of new medicines. They are an important source of bioactive or inspiring molecules. Skepticism can arise from the use of pure isolated compounds if we consider that plants have a mixture of several bioactive molecules that can synergize the biological effects. However, mixtures can also be developed, and the knowledge of their composition will allow for the optimization of its effect, not only against the disease but also on the patient. The authors of the current editorial hope that this special issue stimulates further research, in particular, research involving clinical trials.
